# Altered Resting-State functional connectivity in the anterior and posterior hippocampus in Post-traumatic stress disorder: The central role of the anterior hippocampus

**DOI:** 10.1016/j.nicl.2023.103417

**Published:** 2023-04-28

**Authors:** Mohammad Chaposhloo, Andrew A. Nicholson, Suzanna Becker, Margaret C. McKinnon, Ruth Lanius, Saurabh Bhaskar Shaw, for the Alzheimer's Disease Neuroimaging Initiative

**Affiliations:** a Department of Psychology, Neuroscience & Behaviour, McMaster University, Hamilton, Ontario, Canada; b Department of Psychiatry and Behavioural Neurosciences, McMaster University, Hamilton, Ontario, Canada; i Department of Medical Biophysics, Western University, London, Ontario, Canada; j Atlas Institute for Veterans and Families, Institute of Mental Health Research, University of Ottawa, Royal Ottawa Hospital, Ottawa, Ontario, Canada; k School of Psychology, Faculty of Social Sciences, University of Ottawa, Ottawa, Ontario, Canada; c Vector Institute for Artificial Intelligence, Toronto, Ontario, Canada; d Homewood Research Institute, Guelph, Ontario, Canada; e Mood Disorders Program, St. Joseph’s Healthcare, Hamilton, Ontario, Canada; f Department of Psychiatry, Western University, London, Ontario, Canada; g Department of Neuroscience, Western University, London, Ontario, Canada; h Imaging Division, Lawson Health Research Institute, London, Ontario, Canada

**Keywords:** PTSD, Hippocampus, Functional connectivity, Resting-state fMRI, Graph theory, Hub

## Abstract

**Background:**

Post-traumatic stress disorder can be viewed as a memory disorder, with trauma-related flashbacks being a core symptom. Given the central role of the hippocampus in autobiographical memory, surprisingly, there is mixed evidence concerning altered hippocampal functional connectivity in PTSD. We shed light on this discrepancy by considering the distinct roles of the anterior versus posterior hippocampus and examine how this distinction may map onto whole-brain resting-state functional connectivity patterns among those with and without PTSD.

**Methods:**

We first assessed whole-brain between-group differences in the functional connectivity profiles of the anterior and posterior hippocampus within a publicly available data set of resting-state fMRI data from 31 male Vietnam war veterans diagnosed with PTSD (mean age = 67.6 years, sd = 2.3) and 29 age-matched combat-exposed male controls (age = 69.1 years, sd = 3.5). Next, the connectivity patterns of each subject within the PTSD group were correlated with their PTSD symptom scores. Finally, the between-group differences in whole-brain functional connectivity profiles discovered for the anterior and posterior hippocampal seeds were used to prescribe post-hoc ROIs, which were then used to perform ROI-to-ROI functional connectivity and graph-theoretic analyses.

**Results:**

The PTSD group showed increased functional connectivity of the anterior hippocampus with affective brain regions (anterior/posterior insula, orbitofrontal cortex, temporal pole) and decreased functional connectivity of the anterior/posterior hippocampus with regions involved in processing bodily self-consciousness (supramarginal gyrus). Notably, decreased anterior hippocampus connectivity with the posterior cingulate cortex/precuneus was associated with increased PTSD symptom severity. The left anterior hippocampus also emerged as a central locus of abnormal functional connectivity, with graph-theoretic measures suggestive of a more central hub-like role for this region in those with PTSD compared to trauma-exposed controls.

**Conclusions:**

Our results highlight that the anterior hippocampus plays a critical role in the neurocircuitry underlying PTSD and underscore the importance of the differential roles of hippocampal sub-regions in serving as biomarkers of PTSD. Future studies should investigate whether the differential patterns of functional connectivity stemming from hippocampal sub-regions is observed in PTSD populations other than older war veterans.

## Highlights

• Hyperconnectivity observed between the anterior hippocampus and affective brain areas in PTSD.
• Anterior hippocampus-PCC/precuneus connectivity and CAPS scores are anti-correlated.
• Hippocampus hypo-connected with areas involved in bodily self-consciousness.
• The anterior hippocampus gains a hub-like role in PTSD, revealed by graph-theoretic measures.

## 1 Introduction

Post-traumatic Stress Disorder (PTSD) is a psychiatric condition resulting from exposure to one or more traumatic events (Yehuda et al., 2015). It affects a considerable portion of the population; as of 2008, it was estimated that 9.2% of Canadians had been diagnosed with PTSD at some point during their lives (Van Ameringen et al., 2008). PTSD leads to involuntary, intrusive, and vivid re-experiencing of traumatic memories (i.e., “*flashbacks”*) (Brewin, 2014), intense anxiety, hypervigilance even when no apparent threat is present, and chronic unfavourable changes in cognition and mood (American Psychiatric Association, 2013, Harricharan et al., 2021). Individuals with PTSD may also experience more general memory deficits, including impaired voluntary recall of “ordinary” episodic memories of the trauma (Brewin, 2014), deficiencies in verbal declarative (Bremner et al., 2004) and working memory (Vasterling et al., 2002), over-generalization of fear responses (Brown et al., 2013), and failure to employ contextual information to identify real threats (Garfinkel et al., 2014).

One core component of the episodic memory system is the hippocampus (Scoville and Milner, 1957, Squire, 1986), which is involved in autobiographical memory and episodic future thinking (Okuda et al., 1998, Szpunar et al., 2007), spatial memory, planning and navigation (for a review, see Burgess et al., 2001a), emotional memory (Kim & Fanselow, 1992), emotion regulation (Herman et al., 1989), and encoding of context during fear conditioning (Rudy & Matus-Amat, 2005). Incontrovertibly, the hippocampus has a unique role in forming coherent memories of complex events, by associating multiple elements of an event (such as multisensory information, location, emotion and time) and binding them together (Horner & Burgess, 2013). Therefore, it is unsurprising that the hippocampus has been implicated in the neuropathology of PTSD (Rauch et al., 2006, Shin et al., 2006).

### 1.1 The case for hippocampal dysfunction in PTSD

Hippocampal-related abnormalities are linked to some PTSD symptoms, such as intrusive trauma memories, impaired retrieval of trauma-related details and over-generalization of fear responses (Brewin, 2014, Kheirbek et al., 2012). Specifically, hippocampal inactivity may underlie the overgeneralization of conditioned fear in PTSD (Kaczkurkin et al., 2017). Moreover, hippocampal volume reductions have been observed in PTSD (Bremner et al., 1995, Gurvits et al., 1996), and smaller hippocampal volume may be a risk factor for developing PTSD following a traumatic event (Gilbertson et al., 2002).

Yet another indication of altered hippocampal function in PTSD is the evidence of PTSD-linked changes in large-scale intrinsic brain networks. Three major intrinsic brain networks have been identified within the widely influential *triple network model* (Menon, 2011): the default mode network (DMN), salience network (SN), and central executive network (CEN; also known as the frontoparietal network (FPN)). These networks play a significant role in behaviour and cognition through interactions among them, and abnormalities within and between these networks could be attributed to various psychopathologies (Menon, 2018). The DMN primarily consists of the hippocampus, medial prefrontal cortex (mPFC), posterior cingulate cortex (PCC) and precuneus, and in healthy individuals, it is predominantly active during wakeful rest (Greicius et al., 2003, Raichle et al., 2001), autobiographical memory (AM) retrieval (for a *meta*-analysis, see Svoboda et al., 2006 and future thinking (Addis et al., 2007); moreover, the DMN couples with the SN during AM retrieval (Shaw et al., 2021). The DMN is also involved in self-related mentation, such as mind-wandering, personal introspection, spatial planning and navigation (Burgess et al., 2001b, Spreng et al., 2009, Spreng and Grady, 2010). Importantly, the DMN appears highly dysregulated in PTSD (Koch et al., 2016), as evidenced by decreased within-DMN functional connectivity (Patel et al., 2012, Sripada et al., 2012b), which may underlie PTSD symptoms such as intrusive memories, avoidance (Akiki et al., 2017), deficient autobiographical memory (Menon, 2011), and the loss of a sense of self, exemplified by statements such as I am not me anymore'' following trauma (Foa et al., 1999). These changes in DMN connectivity may be partly explained by an underlying alteration in hippocampal functional connectivity, given its central role in episodic memory (Joshi et al., 2020). Notably, in those with PTSD, the DMN is more strongly coupled with the SN (Akiki et al., 2017, Joshi et al., 2020, Sripada et al., 2012b), comprised of the amygdala, anterior insula, dorsal anterior cingulate cortex (dACC), and temporal pole (TP). Abnormal connectivity has also been observed between other SN regions and brain regions within the innate alarm system (IAS) (Lanius et al., 2017), potentially impacting the functional roles of the SN in detecting salient external stimuli and internal events (Menon, 2011), switching between the DMN and CEN according to task demands (Shaw et al., 2021), and integrating multisensory information with affect and emotions to facilitate an embodied sense of self (Harricharan et al., 2021, Lanius et al., 2020, Nicholson et al., 2020).

One striking aspect of PTSD trauma memories is their firm grounding in sensory-motor representations (Van der Kolk & Fisler, 1995), such as flashbacks accompanied by re-experiencing of pain (for a report of one such individual, see Whalley et al., 2007). One study found that the somatosensory-motor network (SMN), comprised of the pre- and post-central gyri (primary motor cortex and somatosensory cortex, respectively), the primary sensory cortices, and the supplementary motor area (SMA), undergoes a within-network decrease in functional connectivity in those with PTSD, especially in the somatosensory cortex (Shang et al., 2014), which is consistent with catastrophic, fearful orientation to somatic signals in PTSD (Tsur et al., 2018). Conversely, hyperconnectivity between the posterior DMN and SMN in PTSD is consistent with symptoms such as involuntary re-experiencing of, vivid sensory-motor imprints of the original traumatic memory (Kearney et al., 2023). Based on these findings, it is reasonable to hypothesize that PTSD may involve abnormal connectivity between the hippocampus and SMN.

Those with PTSD commonly manifest impaired suppression of flashbacks, which has been at least partially attributed to decreased prefrontal activity. The prefrontal cortex is involved in emotion regulation, decision making, fear extinction and retention of extinction (for reviews of prefrontal involvement in the neurocircuitry of PTSD, see Harricharan et al., 2021, Shin et al., 2006). Prefrontal hypoactivation leads to an inability to exert top-down inhibition on limbic (e.g., amygdala) and brainstem (e.g., periaqueductal gray) regions (Nicholson et al., 2017), potentially leaving those brain areas over-activated in response to emotional cues, irrespective of their trauma relevance (Admon et al., 2013a). Consequently, in the absence of adequate top-down prefrontal control, bottom-up subcortical processes prevail, with *“raw”* affective internal sensations and external stimuli dominating them (Harricharan et al., 2021). However, studies have produced inconsistent findings on amygdala hyperactivation in PTSD, which could be due to task-related differences and the inclusion (or lack thereof) of individuals with the dissociative subtype (Lee et al., 2021, Sartory et al., 2013, Schulze et al., 2019, Stark et al., 2015, Suarez-Jimenez et al., 2020, Thome et al., 2019)(we further consider the dissociative sub-type in the Discussion). The insula and orbitofrontal cortex (OFC) are other brain areas of relevance in PTSD. Children with PTSD who had self-injurious behaviours exhibited elevated insula and OFC activation levels, and their symptom severity correlated positively with insula activation (Carrion et al., 2008). The above evidence raises the question of whether altered hippocampal functional connectivity with structures including the prefrontal cortex, insula and OFC may arise in PTSD.

### 1.2 Distinct functional roles of the anterior and posterior hippocampus

Considering the evidence reviewed so far, it is reasonable to predict that the hippocampus might exhibit altered functional connectivity with other brain areas in those with PTSD. However, findings regarding such alterations are mixed. For example, hippocampal-prefrontal functional connectivity has repeatedly been shown to be decreased in PTSD relative to controls (Heyn et al., 2019, Jin et al., 2014) and relative to exposure therapy recipients (Zhu et al., 2018), and some studies reported decreased functional connectivity between the hippocampus and the amygdala (Sripada et al., 2012a). However, several others reported no functional connectivity differences in the hippocampus in PTSD vs. controls (Brown et al., 2014, Nicholson et al., 2015, Rabinak et al., 2011).

The discrepant reports of altered hippocampal connectivity in PTSD may result from seed-based fMRI studies treating the hippocampus as a single structure (e.g., Carrion et al., 2010, and ignoring potentially crucial functional differences along its longitudinal axis. Though ongoing debate persists regarding precise functional roles of the anterior versus posterior hippocampi (for reviews, see, e.g., Fanselow and Dong, 2010, Poppenk et al., 2013, Strange et al., 2014), human imaging research has increasingly focused on investigating this important question. In healthy humans, evidence indicates greater posterior than anterior hippocampal functional connectivity with the PCC, precuneus (Chen and Etkin, 2013, Poppenk and Moscovitch, 2011), and parahippocampal cortex (Dalton et al., 2019, Libby et al., 2012), while the anterior portion is more functionally connected to perirhinal cortex (Dalton et al., 2019, Libby et al., 2012). Consistent with this evidence from resting-state functional connectivity studies, task-based fMRI studies indicate that the posterior hippocampus has greater activation in spatial tasks requiring precise spatial representations, while the anterior portion is more involved in tasks requiring less detailed contextual information (Brunec et al., 2018, Evensmoen et al., 2013, Evensmoen et al., 2015, Nadel et al., 2013). Accordingly, the predominant view amongst cognitive neuroscientists is that the anterior portion is more heavily involved in gist-like, schematic, or coarse-scaled contextual representations while the posterior portion is more heavily involved in finely detailed spatial representations (Poppenk et al., 2013, Zeidman and Maguire, 2016) (although for a different view, see Dandolo & Schwabe, 2018), where memory recall among those with PTSD has been associated more heavily with the former form of memory (Hayes et al., 2011).

While the above view of the anterior hippocampus as being crucial for schematic representations has considerable empirical support, this view ignores the wealth of convergent evidence from both human and non-human animal studies for a broader role for this region in emotional and stress-related functions. For example, in humans, the anterior subiculum is more heavily functionally connected to the ventral striatum, midbrain, and amygdala (Chase et al., 2015, Kahn and Shohamy, 2013); similarly, in non-human primates, the anterior hippocampus is more connected to emotional and stress-related neural circuitry, including the amygdala (Aggleton, 1986, Wang and Barbas, 2018), the insula (Pribram & Maclean, 1953), and the limbic prefrontal circuitry (Barbas and Blatt, 1995, Carmichael and Price, 1995). Similarly, task-based fMRI studies in humans reveal that the anterior is more activated than the posterior hippocampus in emotional memory tasks (Murty et al., 2011), high state anxiety (Satpute et al., 2012) and goal-directed spatial decision making (Viard et al., 2011). Moreover, in humans with epilepsy, direct recordings in the amygdala and the anterior hippocampus revealed synchronized Beta-frequency activity between these areas during fear memory retrieval (Wang et al., 2020) and greater low-frequency coupling of these areas during processing of fearful faces vs. neutral landscape stimuli (Zheng et al., 2017).

Corresponding to these differences in anatomical and functional connectivity, cellular recording studies in rodents lend more specific evidence as to the type of information encoded along the longitudinal axis of the hippocampus, where in rodents, the long axis is in the dorsal–ventral direction, corresponding to the posterior-anterior direction in primates. In rodents, granule cells in the ventral dentate gyrus suppress intrinsic anxiety without impacting contextual learning (Kheirbek et al., 2013). Moreover, the dorsal CA1 is highly populated by place cells, while the ventral CA1 is dominated by “anxiety cells”, triggered by being in anxiogenic environments and involved in avoidance behaviour (Jimenez et al., 2018). Furthermore, synapses in dorsal CA1 are particularly vulnerable to short and concurrent stress compared to ventral CA1 (Maras et al., 2014), suggesting its sensitivity to psychopathologies such as PTSD, which could render the animal overly reliant upon the ventral hippocampus for memory functions. Interestingly, the posterior hippocampus shows reduced volume in PTSD (Bonne et al., 2008). Thus, when one considers all the evidence across species, it is apparent that the differences between anterior and posterior hippocampal functions in humans go beyond different spatial scales of information representation. Instead, the anterior portion may be more specialized to support detailed memories for the emotional component of events.

Considering the evidence discussed above, we hypothesize that in humans with PTSD, there may be differential abnormal functional connectivity between the anterior versus posterior hippocampus and areas implicated in the neurocircuitry of PTSD, including prefrontal, parietal, and insular cortices. Moreover, investigating the functional connectivity patterns of the anterior and posterior hippocampus separately could have implications for a prominent view of PTSD, the *Dual Representation Theory of PTSD* (Brewin, 2014, Brewin et al., 2010), which proposes that the hippocampus is not appropriately involved in encoding and retrieval of trauma memories, a topic we return to in the discussion.

To the best of our knowledge, only four prior studies have examined the differential resting-state functional connectivity profiles of the anterior and posterior hippocampus in PTSD. Of those four, two studies (Lazarov et al., 2017, Malivoire et al., 2018) employed ROI-to-ROI analyses within narrow pre-defined subsets of regions rather than whole-brain functional connectivity analyses. Lazarov et al., (2017) found that in PTSD, the posterior hippocampus shows increased functional connectivity (reported as decreased negative connectivity) with the precuneus, as well as different functional connectivity patterns for the anterior versus posterior hippocampus among controls but not among those with PTSD (Lazarov et al., 2017). Additionally, increased functional connectivity was found between the posterior hippocampus and PCC in the PTSD group (Malivoire et al., 2018). However, given the aforementioned evidence of widespread brain areas pathologically affected by PTSD that extend well beyond the nodes prescribed by the triple network model, directly assessing functional connectivity via ROI-to-ROI analysis in a restricted set of ROIs may hinder detection of critical changes. Two studies that we are aware of analyzed whole-brain functional connectivity with the anterior vs posterior hippocampus. One such study obtained results in the opposite direction to those of Lazarov et al. and Malivoire et al., i.e., decreased posterior hippocampus functional connectivity with the precuneus and PCC (Chen & Etkin, 2013). Unfortunately, this study was limited by the relatively small sample size of the PTSD group (17 participants). Finally, the fourth study did not include a control group (Jung & Kim, 2020), limiting its ability to detect PTSD-linked functional connectivity changes relative to healthy controls. To resolve the above discrepant findings in the literature, a follow-up study is warranted, incorporating a control group and a much larger sample size, utilizing a data-driven approach to assess whole-brain differences in anterior vs. posterior hippocampal functional connectivity in those with PTSD. Moreover, while previous research has applied graph-theoretical analyses to whole-brain connectivity in PTSD (Suo et al., 2015, Zhu et al., 2019), to our knowledge, no studies have investigated hippocampal connectivity specifically.

Accordingly, in the present study, we performed a seed-based whole-brain functional connectivity analysis, separately seeding the anterior versus posterior hippocampi, followed by post-hoc ROI-to-ROI connectivity analysis on the discovered clusters. This data-driven approach does not limit the functional connectivity analysis to previously defined brain regions, providing the best chance of discovering altered patterns of hippocampal functional connectivity in those with PTSD in an unbiased manner. Based on our current understanding of the unique connectivity profiles of the anterior and posterior hippocampus and considering the previous research reviewed above, we predicted the following:

1. Given the SN’s role in assessing potential threats and identifying salient stimuli, and with hypervigilance and hyperarousal being core symptoms of PTSD, we predicted a functional connectivity increase between the anterior hippocampus and SN nodes. Additionally, considering the greater relevance of the anterior hippocampus to emotion and stress-related functions, we expected it to play a greater role in PTSD, potentially exhibiting stronger rather than weaker functional connectivity with stress-related circuits compared to the posterior hippocampus.
2. We hypothesized that the functional connectivity between the posterior hippocampus and DMN would be diminished in PTSD on the grounds that individuals with PTSD demonstrate impaired episodic memory and internal mentation.
3. Given that those with PTSD exhibit alterations in their sense of body and self, and many therapeutic efforts are geared towards targeting somatic and motor pathways, we expected to observe altered functional connectivity between both the anterior and posterior hippocampus and somatosensory and motor areas.

The present study was undertaken to test the above predictions in a freely available set of resting state fMRI data previously collected from a sample of individuals with PTSD.

## 2 Material and methods

### 2.1 Participants

We utilized a previously collected, open-source set of resting-state fMRI data acquired from male Vietnam War veterans, obtained from the Alzheimer’s Disease Neuroimaging Initiative (ADNI) database (http://adni.loni.usc.edu). The ADNI was launched in 2003 as a public–private partnership, led by Principal Investigator Michael W. Weiner, MD. The primary goal of ADNI was to test whether serial magnetic resonance imaging (MRI), positron emission tomography (PET), other biological markers, and clinical and neuropsychological assessment can be combined to measure the progression of mild cognitive impairment (MCI) and early Alzheimer’s disease (AD). For up-to-date information, see https://www.adni-info.org. The ethics boards of all collaborating sites within ADNI approved the collection of this data set, and all participants provided written informed consent. While the primary focus of ADNI is on AD, a sizeable subset of participants was diagnosed with PTSD without exhibiting symptoms of mild cognitive impairment (MCI) or AD. For the analyses reported here, 60 male, combat-exposed subjects (mean age = 68.3 years, sd = 3.0) were selected, excluding those with MCI, traumatic brain injury or AD. Of those 60, 31 (mean age = 67.6 years, sd = 2.3) were included in the PTSD group, with the inclusion criterion of Clinician-Administered PTSD Scale IV (CAPS-IV, assessed decades after their war exposure) ≥ 50 (average CAPS-IV within the PTSD group = 64.7, sd = 13.3). This inclusion criterion (CAPS > 50) has been extensively used previously to define PTSD groups in neuroimaging analyses (e.g., Harricharan et al., 2020, Rabellino et al., 2015, Terpou et al., 2018). The remaining 29 participants (mean age = 69.1 years, sd = 3.5) were included in the control group (average CAPS-IV = 1.5, sd = 2.9). A Welch’s *t*-test to assess differences in the mean age of the two groups revealed that they were not significantly different (t(48.7351) = −1.9610, p = 0.0556).

### 2.2 Neuroimaging data acquisition and pre-processing

We downloaded all T1-weighted anatomical scans along with corresponding resting-state fMRI scans from the ADNI website, where the details of data acquisition and preliminary pre-processing steps can also be found (https://adni.loni.usc.edu/methods/mri-tool/mri-analysis/). All MRI data were acquired using GE 3T MRI scanners (General Electric Healthcare, Milwaukee, WI). In brief, a T1-weighted anatomical scan was acquired for each participant using IR prepped sagittal 3D SPGR sequence (TI/TR/TE = 400/7.34/3.04 ms, 11 flip angle, 1.2 mm-thick slices of size 256 × 256) along with resting-state fMRI scans with 160 time points (Scanning Sequence: EP/GR, TR = 2.9 ∼ 3.52 s, TE = 30 ms, 3.3 mm-thick slices of size 64 × 64, 48 slices per time point).

fMRI data were pre-processed using SPM12 (Wellcome Centre for Human Neuroimaging, London, UK) and the CONN toolbox (Whitfield-Gabrieli & Nieto-Castanon, 2012) within MATLAB version R2020a (The MathWorks, Inc., Natick, MA, USA). We used the default pre-processing and denoising pipelines within CONN, including realignment and unwarping of the fMRI scans, followed by motion correction using estimates of motion along 12 degrees-of-freedom (3 translation, 3 rotation, 3 first-derivatives of translation, 3 first-derivatives of rotation) as nuisance regressors in a denoising general linear model (GLM). Next, frequency-domain based phase shift slice timing correction (STC) was applied, along with scrubbing of outlier scans detected using ART. A unified segmentation and normalization procedure (Ashburner and Friston, 2005) was then used to normalize the scans to the MNI152 atlas and segment skull, white matter, grey matter and cerebro-spinal fluid (CSF). Potential physiological confounds were minimized by including the average signal from white matter and CSF as nuisance regressors. Finally, spatial smoothing was applied with an 8 mm full-width-at-half-maximum (FWHM) Gaussian kernel, followed by temporal band-pass filtering (0.008–0.09 Hz).

### 2.3 Functional connectivity analysis

Resting-state functional connectivity analyses were performed using the CONN toolbox (Whitfield-Gabrieli & Nieto-Castanon, 2012). The first analysis performed was a seed-to-voxel connectivity analysis while seeding the entire hippocampus, and then the anterior and posterior hippocampus. To perform this analysis, the seed regions of interest (ROIs) for the left and right anterior and posterior hippocampus were acquired from the Brainnetome atlas (Fan et al., 2016). Next, the mean BOLD signal intensity time course was extracted for each seed and for each subject. Then, a whole-brain functional connectivity analysis was performed, where for each subject and each hippocampal ROI, the Fischer-transformed correlation coefficient between the time course of the seed ROI and the time course of every other voxel in the brain was calculated, resulting in a whole-brain map of functional connectivity for each seed ROI and every subject (Bijsterbosch et al., 2017). These maps were then used in a second-level group analysis where we compared the PTSD group against the control group using the PTSD > Control contrast. In addition, we correlated the whole-brain functional connectivity of each seed ROI (used for the seed-to-voxel analysis described above) with the CAPS-IV scores for subjects within the PTSD. These results were corrected for multiple comparisons at the cluster level (Worsley et al., 1996), excluding clusters that did not meet a voxel-discovery threshold of p-uncorrected < 0.001 and a cluster-level p-FDR < 0.05.

To further investigate group differences between hippocampal sub-regions and other parts of the brain, we then performed a post-hoc ROI-to-ROI analysis, where we estimated the functional connectivity between hippocampal seed ROIs and target ROIs, defined using the clusters discovered in the previous seed-to-voxel analysis. In this way, we could investigate the functional connectivity of those brain areas that did not survive correction for multiple tests but showed a trend nevertheless. Target ROIs were defined in a data-driven manner. To do so, we identified clusters of differences in functional connectivity values for each brain region. These clusters may or may not survive multiple comparison corrections. Next, a spherical ROI with a radius of 5 mm was placed in the centre of each cluster using the MarsBaR toolbox (Brett et al., 2002). The post-hoc analysis was designed to further investigate connectivity patterns over restricted brain regions, similar to the network-restricted approach followed by Akiki et al., 2018, and care was taken to minimize Type-1 error (Brooks et al., 2017, Kriegeskorte et al., 2009) by including a wider set of brain regions based on prior PTSD literature. This was performed in lieu of orthogonal contrasts (Kriegeskorte et al., 2009) recommended for reproducibility due to the limited number of experimental conditions available from the publicly available data set used in this study. Furthermore, the risk of limited reproducibility was also mitigated by the use of this publicly available data set that can be independently downloaded and assessed.

### 2.4 Graph-theoretic analysis

Finally, to better understand the global properties of the observed ROI-to-ROI connectivity, we analyzed group differences in graph-theoretic measures. While ROI-to-ROI analyses identify differences in functional connectivity between ROI pairs, graph-theoretic analyses assess the global role of a node (ROI) within the larger group of ROIs, providing a global overview of each node’s functional connectivity profile. For instance (and much to our interest), it can reveal which nodes act as hubs that are heavily (and centrally) connected with many other nodes and can efficiently transfer information between them (Bullmore & Sporns, 2009). “Hubness”, or the hub-like behaviour of a node, is often assessed by measures of centrality (e.g., degree, cost, betweenness centrality; described below), and efficiency (path length and clustering coefficient; for a review of hubness in the context of brain science, see van den Heuvel & Sporns, 2013). To perform the graph-theoretic analyses, we first defined a graph for each participant using the ROIs studied as the nodes, and the ROI-to-ROI connectivity between every pair of nodes as the edges. To allow sensitive between-network comparisons, the graphs were thresholded to only include the top 15% of connections based on their cost (described below). These graphs were then used to estimate several node-based graph theoretic measures; namely,

1. *degree* - an estimate of how connected the current node is, as determined by the number of neighbouring nodes,
2. *cost* (also known as *strength*) - is the weighted form of the degree and gives an estimate of the net connectivity strength. It is determined as the sum of all neighbouring weights, accounting for both the number of edges and their strength,
3. *path length* - quantifies the distance that information has to travel to reach other nodes from the current node. It is determined by the number of edges that constitute the shortest path between two nodes,
4. *node-wise global efficiency* - is an estimate of the efficiency of information transfer from the current node to all other nodes, determined by the average of inverse path lengths leading to a node across the entire graph,
5. *node-wise local efficiency* - is an estimate of the efficiency of information transfer from the current node to nodes it is directly connected to, determined by as the average global efficiency across the sub-graph consisting of only the neighbours of the given node.
6. *clustering coefficient* - is an estimate of how well the neighbours of a node are connected to each other and form a cluster (defined as the number of existing edges between neighbours of a node divided by the total number of possible edges between those same nodes), and
7. *betweenness centrality* - an estimate of how central the node is in the network (defined as the fraction of all shortest paths that a node participates in).

Finally, group differences in the above node-wise graph-theoretic metrics were assessed after FDR-based corrections for multiple comparisons were applied. All analyses were performed using the CONN toolbox 20.b (Whitfield-Gabrieli & Nieto-Castanon, 2012).

## 3 Results

### 3.1 Whole-brain functional connectivity analysis

**Fig. 1:**
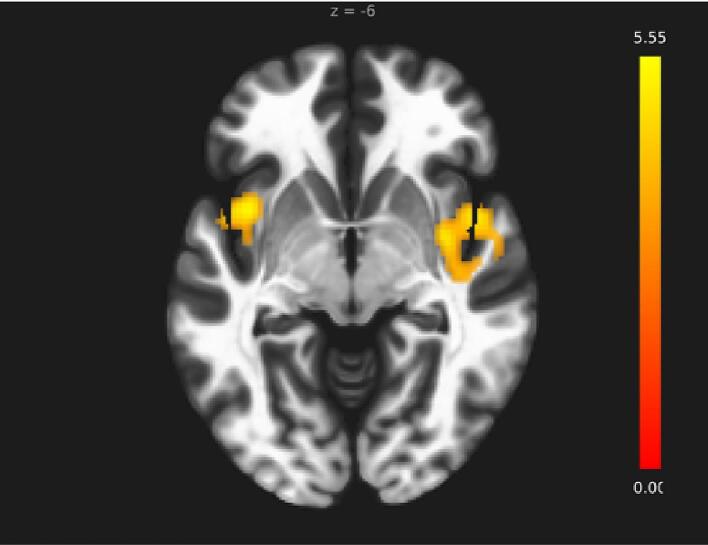
Areas of increased functional connectivity with the left anterior hippocampus. Whole-brain functional connectivity analysis revealed that in the PTSD group, the left anterior hippocampus was significantly more connected to the left anterior insula, right posterior insula, and right temporal pole (areas shown in yellow) as compared to the control group (the colour bar represents T-values). (For interpretation of the references to color in this figure legend, the reader is referred to the web version of this article.)

We started by seeding the entire hippocampus to investigate whether the functional connectivity of the hippocampus with any brain regions differs between the two groups. No significant group differences were found when the seed ROI was the entire hippocampus. We then separately seeded the anterior and posterior hippocampi to examine the group differences along the long axis of the hippocampus. The bilateral posterior hippocampus (pHipp) and right anterior hippocampus (aHipp) exhibited no significant group differences. However, when the seed ROI was the left aHipp, it showed significantly more functional connectivity with the left anterior insula (aIC), right posterior insula (pIC), and right temporal pole (TP) in PTSD compared to the control group (Table 1 and Fig. 1). These previously unreported and novel findings provide the first insight into seed-based whole-brain functional connectivity differences stemming from the aHipp, suggesting a dysfunction in emotion processing circuitry (aHipp) along with affective brain regions (a/pIC and TP).

**Table 1:** Significant clusters that showed increased functional connectivity with the left anterior hippocampus for the PTSD *>* Controls contrast in the whole-brain seed-based functional connectivity analysis. TP: Temporal pole; pIC: Posterior insula; aIC: Anterior insula.

Brain Region	Cluster size	T-statistics	p(FDR)	MNI Coordinates (mm)
R. TP/R. pIC	472	T(58) = 5.11	0.001454	+36–2 −8
L. aIC	281	T(58) = 5.55	0.011783	−38 + 8–8

**Fig. 2:**
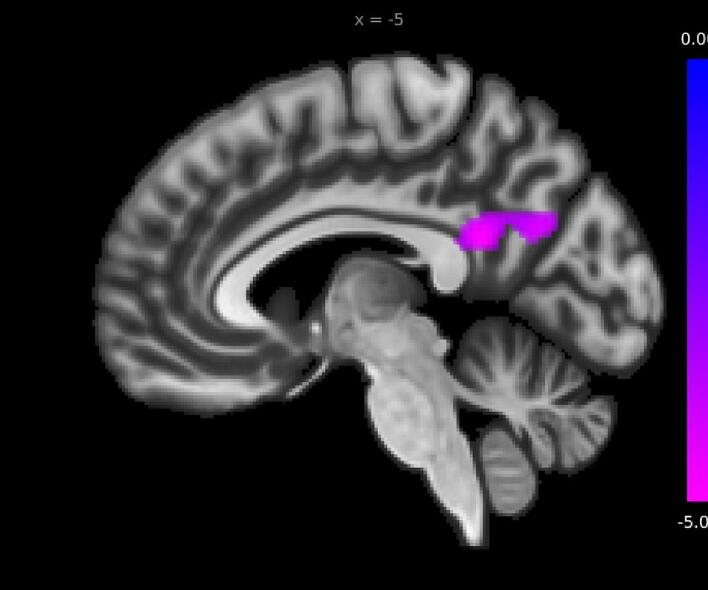
Medial sagittal view of the left hemisphere showing that within the PTSD group, symptoms severity as represented by CAPS scores was negatively correlated with the functional connectivity between the right anterior hippocampus and PCC/precuneus (areas shown in magenta; the colour bar represents T-values). PCC: Posterior cingulate cortex; CAPS: Clinician-Administered PTSD Scale. (For interpretation of the references to color in this figure legend, the reader is referred to the web version of this article.)

The next question we sought to answer was to what degree the functional connectivity of hippocampal subregions correlated with symptom severity in PTSD. Here, the CAPS-IV score provided a suitable and general measure of symptom severity in PTSD. Again, only the aHipp yielded significant results. Unexpectedly, within the PTSD group, the functional connectivity of the right aHipp with PCC and precuneus was negatively correlated with CAPS scores (cluster size = 346, T(29) = -5.07, p-FDR = 0.0009, MNI coordinates (mm) = −6–48 24; Fig. 2). This finding seems to be at odds with our second hypothesis that the pHipp rather than aHipp would show diminished functional connectivity with DMN nodes in those with PTSD. We return to this point later on.

### 3.2 ROI-to-ROI functional connectivity analysis

Based on the whole-brain functional connectivity analysis, 21 target ROIs were manually defined that had differential functional connectivity in PTSD compared to controls. MNI coordinates of these ROIs are listed below (Table 2). Next, we conducted an ROI-to-ROI analysis on these 21 ROIs (see Table 3).

**Table 2:** MNI coordinates of the 21 target ROIs used with the hippocampal ROIs (source ROIs) for the ROI-to-ROI functional connectivity analysis.

MNI coordinates of target ROIs	
Brain Region	Coordinates (mm)
left anterior insula (aIC)	[–39 7 –8]
right anterior insula (aIC)	[37 13 –13]
left posterior insula (pIC)	[–39 –6 −6]
right posterior insula (pIC)	[39 –6 −6]
left temporal pole (TP)	[–47 14 –14]
right temporal pole (TP)	[49 5 –6]
left lateral orbitofrontal cortex (lOFC)	[–29 21 –19]
right lateral orbitofrontal cortex (lOFC)	[35 22 –17]
left periaqueductal gray (PAG)	[–5 –26 −12]
right periaqueductal gray (PAG)	[5 –29 −14]
right anterior superior temporal gyrus (aSTG)	[59 –8 −5]
right posterior superior temporal gyrus (pSTG)	[61 –32 10]
left posterior superior temporal gyrus (pSTG)	[–60 –34 14]
right posterior middle temporal gyrus (pMTG)	[61 –33 −10]
left posterior middle temporal gyrus (pMTG)	[–60 –48 7]
left posterior inferior temporal gyrus (pITG)	[–54 –48 −15]
right angular gyrus	[51 –48 22]
left postcentral gyrus	[–43 –24 60]
left supramarginal gyrus (SMG)	[–55 –24 50]
left precuneus, A7m, medial area 7(PEp)	[–6 –68 49]
right precuneus, A7m, medial area 7(PEp)	[6 –62 46]
ventromedial prefrontal cortex (vmPFC)	[–3 40 0]

**Table 3:** The results of the post-hoc ROI-to-ROI functional connectivity analysis between the seed hippocampal ROIs and target ROIs. All the connections were FDR corrected at the cluster level. aHipp: Anterior hippocampus; pHipp: Posterior hippocampus; aIC: Anterior insula; pIC: Posterior insula; TP: Temporal pole; lOFC: Lateral orbitofrontal cortex. pSTG: Posterior superior temporal gyrus. pMTG: Posterior middle temporal gyrus; SMG: Supramarginal gyrus; pITG: Posterior inferior temporal gyrus; vmPFC: Ventromedial prefrontal cortex.

Seed ROI	Target ROI	T-statistics	p(FDR)
Left aHipp	Left aIC Right aIC	*T*(58) = 4.69 *T*(58) = 2.47	*p*-FDR = 0*.*0004 *p*-FDR = 0*.*0396
	Right pIC Left pIC	*T*(58) = 2.99 *T*(58) = 2.82	*p*-FDR = 0*.*0135 *p*-FDR = 0*.*0189
	Right TP Left TP	*T*(58) = 3.96 *T*(58) = 2.46	*p*-FDR = 0*.*0027 *p*-FDR = 0*.*0396
	Right lOFC Left lOFC Right pSTG	*T*(58) = 3.03 *T*(58) = 2.01 *T*(58) = 3.49	*p*-FDR = 0*.*0135 *p*-FDR = 0*.*0900 *p*-FDR = 0.0082
	Left pSTG	*T*(58) = 3.17	*p*-FDR = 0*.*0125
	Left pMTG	*T*(58) = 2.41	*p*-FDR = 0*.*0418
	Right precuneus Left precuneus	*T*(58) = 3.26 *T*(58) = 2.28	*p*-FDR = 0*.*0121 *p*-FDR = 0*.*0522
	Left SMG	*T*(58) = -2.99	*p*-FDR = 0*.*0135
Right aHipp	Right aIC Left aIC	*T*(58) = 2.70 *T*(58) = 2.00	*p*-FDR = 0*.*0391 *p*-FDR = 0*.*1080
	Left TP Right TP	*T*(58) = 2.97 *T*(58) = 2.36	*p*-FDR = 0*.*0246 *p*-FDR = 0*.*0581
	Right lOFC	*T*(58) = 2.39	*p*-FDR = 0*.*0581
	Left pSTG Right pSTG	*T*(58) = 2.94 *T*(58) = 2.49	*p*-FDR = 0*.*0246 *p*-FDR = 0*.*0581
	Left pMTG	*T*(58) = 3.49	*p*-FDR = 0*.*0242
	Left pITG	*T*(58) = 3.19	*p*-FDR = 0*.*0246
	Right precuneus Left precuneus	*T*(58) = 2.35 *T*(58) = 2.21	*p*-FDR = 0*.*0581 *p*-FDR = 0*.*0728
	Left SMG	*T*(58) = −2.97	*p*-FDR = 0*.*0246
Left pHipp	Right lOFC	*T*(58) = 2.72	*p*-FDR = 0*.*0742
	Right precuneus	*T*(58) = 2.78	*p*-FDR = 0*.*0742
	Right pSTG	*T*(58) = 3.18	*p*-FDR = 0*.*0615
	Left pMTG	*T*(58) = 2.49	*p*-FDR = 0*.*0845
	Right angular gyrus	*T*(58) = 2.48	*p*-FDR = 0*.*0845
	vmPFC	*T*(58) = −2.18	*p*-FDR = 0*.*1428
Right pHipp	Right lOFC	*T*(58) = 2.19	*p*-FDR = 0*.*1421
	Right precuneus	*T*(58) = 2.96	*p*-FDR = 0*.*0562
	Right pSTG	*T*(58) = 2.62	*p*-FDR = 0*.*0725
	Right angular gyrus	*T*(58) = 2.82	*p*-FDR = 0*.*0562
	Left postcentral gyrus	*T*(58) = −2.37	*p*-FDR = 0*.*1086
	Left SMG	*T*(58) = −2.88	*p*-FDR = 0*.*0562

This approach allowed us to more carefully examine functional connectivity differences between the brain regions that were observed to differ in the seed-based functional connectivity analysis in PTSD, increasing statistical power while correcting for multiple comparisons (Poldrack, 2007). In addition to these 21 ROIs, an ROI for the amygdala was added from the Harvard-Oxford atlas provided with the CONN toolbox. Here, it is important to note that although we did not observe any group differences in hippocampus-amygdala functional connectivity in the whole-brain seed-based analysis, the extensive literature surrounding abnormal functional connectivity of these two regions in PTSD (especially between them) (Heyn et al., 2021, McIntosh et al., 2022, Sripada et al., 2012a, Zhang et al., 2016, Zhu et al., 2018) justifies the inclusion of the amygdala in our analysis. Likewise, while we did not observe any group differences in functional connectivity between the ventromedial prefrontal cortex (vmPFC) and the hippocampus, many theories of PTSD regard vmPFC as a key region involved in the symptomatology of PTSD (Shin et al., 2006), motivating the inclusion of the vmPFC in our analysis. The ROI for vmPFC was acquired from a recent study carried out in our lab (Shaw et al., 2021). In the following paragraphs, we summarize the results of functional connectivity analyses between these ROIs and the hippocampal ROIs acquired from the Brainnetome atlas (Fan et al., 2016).

### 3.3 Anterior hippocampus

**Fig. 3:**
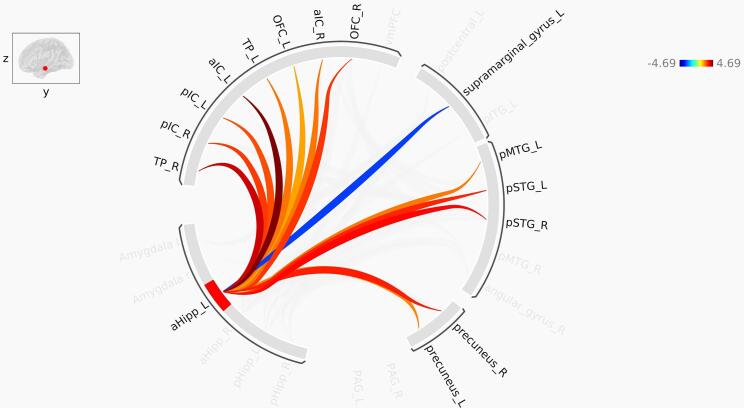
Pathways identified in ROI-to-ROI functional connectivity analysis of the left anterior hippocampus. Red lines represent increased functional connectivity, and blue lines indicate decreased functional connectivity in PTSD compared to control. aHipp: Anterior hippocampus; aIC: Anterior insula; pIC: Posterior insula; TP: Temporal pole; OFC: orbitofrontal cortex; pSTG: Posterior superior temporal gyrus; pMTG: Posterior middle temporal gyrus. The color bar represents T-values. (For interpretation of the references to color in this figure legend, the reader is referred to the web version of this article.)

**Fig. 4:**
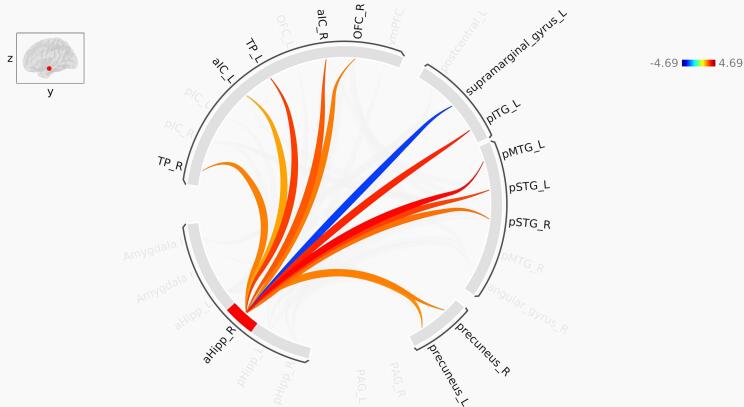
Pathways identified in ROI-to-ROI functional connectivity analysis of the right anterior hippocampus. Red lines represent increased functional connectivity, and blue lines indicate decreased functional connectivity in PTSD compared to control. aHipp: Anterior hippocampus; aIC: Anterior insula; TP: Temporal pole; OFC: orbitofrontal cortex. pSTG: Posterior superior temporal gyrus; pMTG: Posterior middle temporal gyrus; pITG: Posterior inferior temporal gyrus. The color bar represents T-values. (For interpretation of the references to color in this figure legend, the reader is referred to the web version of this article.)

Bilateral aHipp was more connected to bilateral anterior insula (aIC) and bilateral temporal pole (TP) in the PTSD group relative to controls. Additionally, the left aHipp was more connected to bilateral pIC and bilateral lOFC and the right aHipp was more connected to the right lOFC in the PTSD group relative to controls. It is noteworthy that these are all considered to be affective brain regions. Other brain areas that exhibited greater functional connectivity with the aHipp in PTSD included the posterior portions of the superior, medial and inferior temporal gyrus (pSTG, pMTG and pITG, respectively), areas that support unisensory and multisensory processing. Specifically, we observed increased functional connectivity between the bilateral aHipp and bilateral pSTG and left pMTG. Additionally, the right aHipp was more connected to the left pITG in PTSD relative to controls. We also observed greater bilateral aHipp functional connectivity with bilateral precuneus, a key DMN node important for mental imagery, among other functions (Byrne et al., 2007, Cavanna and Trimble, 2006). The greater functional connectivity of aHipp with these areas critical for visual and auditory perception and mental imagery is consistent with the symptomatology of flashbacks, which may also include auditory components (Hackmann et al., 2004). In contrast to these findings of greater functional connectivity, the left supramarginal gyrus (SMG) was less connected to bilateral aHipp in PTSD relative to the control group. Interestingly, the SMG is implicated in bodily self-consciousness (Blanke et al., 2015), and in PTSD, there have been reports of altered bodily representation in *peri*-personal space (Rabellino et al., 2020) and sense of body ownership (Rabellino et al., 2018a). In summary, the aHipp exhibited elevated functional connectivity with many brain regions involved in affective, visual, auditory and multi-sensory processing and mental imagery, whereas it showed less functional connectivity with areas involved in bodily self-consciousness (Fig. 3, Fig. 4).

### 3.4 Posterior hippocampus

**Fig. 5:**
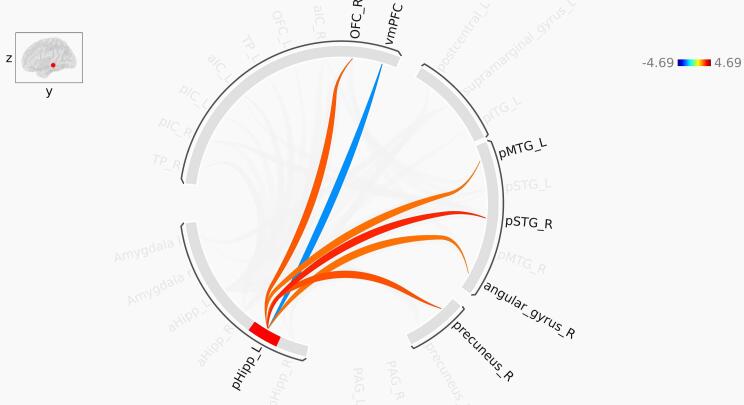
Pathways identified in ROI-to-ROI functional connectivity analysis of the left posterior hippocampus. Red lines represent increased functional connectivity, and blue lines indicate decreased functional connectivity in PTSD compared to control. pHipp: Posterior hippocampus; OFC: Orbitofrontal cortex; pSTG: Posterior superior temporal gyrus; pMTG: Posterior middle temporal gyrus; vmPFC: Ventromedial prefrontal cortex. The color bar represents T-values. (For interpretation of the references to color in this figure legend, the reader is referred to the web version of this article.)

**Fig. 6:**
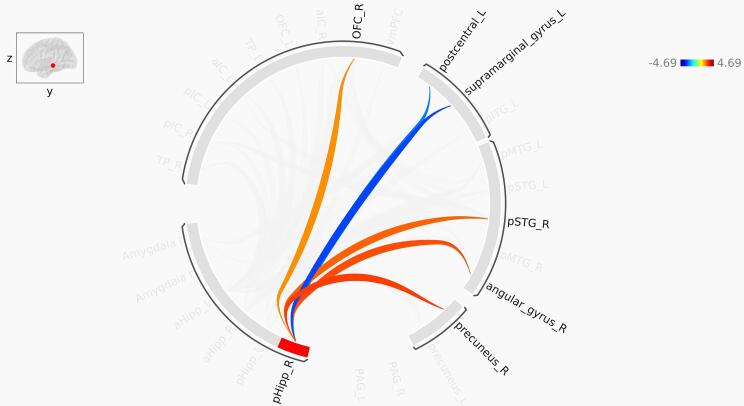
Pathways identified in ROI-to-ROI functional connectivity analysis of the right posterior hippocampus. Red lines represent increased functional connectivity, and blue lines indicate decreased functional connectivity in PTSD compared to control. pHipp: Posterior hippocampus; OFC: Orbitofrontal cortex; pSTG: Posterior superior temporal gyrus. The color bar represents T-values. (For interpretation of the references to color in this figure legend, the reader is referred to the web version of this article.)

The ROIs showing increased functional connectivity with bilateral pHipp in PTSD, compared to controls, were the right lOFC, right precuneus, right pSTG, and right angular gyrus. Furthermore, the left pHipp had elevated functional connectivity with left pMTG in PTSD, relative to controls. On the other hand, the left pHipp was less connected to vmPFC, while the right pHipp was less connected to the left postcentral gyrus and the left SMG in PTSD relative to controls. The decreased functional connectivity between the right pHipp and the left postcentral gyrus is quite interesting since the latter is the loci of the primary somatosensory cortex, and as noted earlier, bodily representation in PTSD is often compromised (Fig. 5, Fig. 6).

Taken together, the above findings indicate that the pHipp exhibits significantly fewer abnormal connections with affective ROIs (insula, TP, and lOFC), as compared to the aHipp. The pHipp also showed decreased functional connectivity with areas involved in somatosensation. Surprisingly, neither the anterior nor posterior hippocampus showed any group difference in functional connectivity with the amygdala, in contrast to previous findings in the literature (Sripada et al., 2012a, Zhu et al., 2018). The increased functional connectivity with the precuneus and various regions of the temporal gyri is a recurring theme for both the anterior and posterior hippocampus, consistent with the multisensory imagery of flashbacks (Hackmann et al., 2004, Van der Kolk and Fisler, 1995).

### 3.5 Graph-theoretic analysis

As the final step in our analyses, we examined our set of ROIs and the functional connectivity between them for node-wise group differences from a graph-theoretic perspective (Rubinov & Sporns, 2010) in order to see whether PTSD is associated with changes in the topology of global connectivity, and if so, which nodes are at the center of these changes. Interestingly, only the left aHipp showed significant group differences between the PTSD and control groups. It displayed a lower average path length (T(58) = −4.00, p-FDR = 0.005) in the PTSD group relative to controls, indicating that the paths leading to the left aHipp are shorter in those with PTSD compared to controls. Similarly, the left aHipp had a higher node-wise global efficiency (T(58) = 4.45, p-FDR = 0.001), cost (T(58) = 4.04, p-FDR = 0.004) and degree (T(58) = 4.04, p-FDR = 0.004) compared to the control group. These results indicate that in PTSD, connections leading to the left aHipp become significantly more numerous and stronger (manifested in increased degree and cost), which in turn gives rise to shorter paths leading to the left aHipp. The resultant effect of these changes is greater efficiency of information flow between these ROIs, via the aHipp (greater node-wise global efficiency). However, the left aHipp failed to show group differences for local efficiency (T(58) = −2.39,p-uncorrected = 0.02), clustering coefficient (T(58) = −1.07, p-uncorrected = 0.29), and betweenness centrality (T(58) = 1.50, p-uncorrected = 0.14). Collectively, these group differences highlight an increase in hub-like properties of the aHipp in those with PTSD as compared to trauma-exposed controls, potentially indicating an adaptive, central role of the aHipp in driving activity in a network of PSTD-relevant brain regions.

## 4 Discussion

This study examined the functional connectivity profile of the anterior and posterior hippocampus in individuals with PTSD and in trauma-exposed controls, using both whole-brain and post-hoc ROI-to-ROI approaches. The whole-brain seed-based analysis revealed no significant group differences when either the entire hippocampus or the posterior hippocampus (pHipp) was used as the seed ROI. In contrast, the anterior hippocampus (aHipp) was significantly more connected to affective brain regions (i.e., anterior and posterior insula and temporal pole) in PTSD compared to controls. Similarly, our post-hoc ROI-to-ROI analysis revealed more abnormal connections for the aHipp than pHipp in those with PTSD. Critically, our graph-theoretic analyses revealed that the left aHipp exhibited more hub-like properties in PTSD compared to the control group, showing lower average path length and higher global efficiency and degree. These results add to a body of evidence for increased global and local efficiency and centrality within nodes of the DMN and SN in those with PTSD, suggesting an adaptation (Suo et al., 2015, Zhu et al., 2019). Moreover, our graph-theoretic results align with a recent study that identified the entire hippocampus as a structural hub within the adult human brain (Oldham & Fornito, 2019). Here, our novel finding that the aHipp (and not the pHipp) exhibits an increase in its hubness likely signals it acquiring a more central role in communication within the brain, providing a more efficient integration of memory processes with other brain regions in PTSD relative to controls, perhaps in compensation for a possible deficit in posterior hippocampal functions, including detailed episodic retrieval. Speculatively, this could also indicate aHipp, a hippocampal sub-region linked to more emotional and schematic memory representations, taking on a more dominant role in controlling memory retrieval processes in those with PTSD, who are known to exhibit overgeneralization in memory retrieval (Schönfeld et al., 2007). On balance, the aHipp appears to be hyperconnected to emotional and other brain regions and may play a more central hub-like role in PTSD as compared to the pHipp.

### 4.1 Anterior hippocampus: the main player in PTSD

#### 4.1.1 Insula

Our most robust finding was increased functional connectivity between the aHipp and anterior/posterior insula in PTSD (Fig. 1). The anterior insula is a major hub in the SN, involved in network switching and predisposing attention to salient interoceptive sensations and exteroceptive stimuli (Menon, 2011). Previous research has yielded mixed results regarding hippocampal-SN connectivity in PTSD; some studies reported hyperconnectivity (Harricharan et al., 2020, Sripada et al., 2012b), while others found hypoconnectivity (Breukelaar et al., 2021), or no differences in PTSD compared to controls (Brown et al., 2014). Our analyses, incorporating separate aHipp and pHipp seeds, offer a resolution to these discrepant findings, as we showed increased aHipp, but not pHipp functional connectivity with the anterior insula, consistent with the anterior insula’s role in salience detection (Downar et al., 2002, Wiech et al., 2010), which becomes abnormal in PTSD (Russman Block et al., 2020). Abnormal salience processing could lead to benign stimuli being identified as threatening, accounting for persistent hypervigilance and hyperarousal in individuals with PTSD (Sripada et al., 2012b, Viard et al., 2019). Notably, the extensive (structural) connectivity between the insula and hippocampus (Ghaziri et al., 2018) contributes to encoding of negative stimuli (Chang and Yu, 2019, Tsukiura et al., 2013). Moreover, presentation of trauma-related cues leads to increased insula activation (Etkin & Wager, 2007), and hyperactivation of the right anterior insula, which correlates positively with state re‐experiencing symptoms (Hopper et al., 2007). The anterior and posterior insula work together to accomplish important salience roles. In healthy adults, the input from the brainstem and thalamus to the posterior insula contains information about raw affective and interoceptive states, in addition to exteroceptive sensory information, which is then passed to the anterior insula where saliency of this information is assessed (Koch et al., 2016, Uddin, 2014). Here, the anterior insula is thought to “translate” this information for the prefrontal cortex, which participates in multisensory integration and emotion regulation (Harricharan et al., 2021). Thus, abnormalities in anterior insula-aHipp functional connectivity could be one of the factors underlying the misattribution of emotional salience to otherwise ordinary events in those with PTSD (Menon, 2011) and their inability to regulate emotions. Specifically, increased functional connectivity between the aHipp and the anterior insula may reduce the hippocampus’ ability to discern non-threatening circumstances (Akiki et al., 2017), which could account for amplified threat processing, hypervigilance and anxiety in individuals with PTSD (Koch et al., 2016). However, heightened threat processing observed in PTSD may also result from bottom-up drive initiated by regions of the innate alarm system such as the periaqueductal grey with less top-down PFC control (Nicholson et al., 2017).

#### 4.1.2 Temporal pole

In addition, we observed increased functional connectivity between the aHipp and temporal pole (TP). The TP has extensive connections with the amygdala and orbitofrontal cortex, and is part of the SN (Menon, 2011). It has been implicated in various functions, such as language processing, visual recognition, autobiographical episodic memories, and socio-emotional processing (Herlin et al., 2021, Olson et al., 2007). Notably, several neuroimaging studies implicated the right TP in emotional situations (Herlin et al., 2021), such as retrieval of emotional autobiographical memories (Dolan et al., 2000, Reiman et al., 1997) or watching emotional movies (Lane et al., 1997, Reiman et al., 1997). War veterans with PTSD showed higher left TP activation when viewing war-related photos compared to combat-exposed controls, with war-related pictures inducing even more TP activation versus neutral photos (Dunkley et al., 2019). Similarly, a PET study involving recalling traumatic autobiographical memories vs. neutral events found that the traumatic condition evoked higher activation in the anterior TP, with the extent of this hyperactivation being even greater in the PTSD group (Shin et al., 1999). Therefore, the increased functional connectivity between the aHipp and the right TP could partially account for the over-representation of traumatic memories in PTSD and hyper-vigilance symptoms. However, the evidence implicating the TP in functional connectivity analysis of PTSD is limited and more research is warranted to elucidate the role of the TP in PTSD.

#### 4.1.3 PCC/Precuneus

Regarding the PCC and precuneus, we did not find any group difference in whole-hippocampal-PCC functional connectivity; however, when separately assessing a/pHipp functional connectivity, we observed elevated coupling with the precuneus in the PTSD group, especially in the aHipp (in contrast to previous findings of reduced precuneus-whole-hippocampal functional connectivity in PTSD (Akiki et al., 2018, Chen and Etkin, 2013, Miller et al., 2017, Viard et al., 2019)). Moreover, decreased functional connectivity between the aHipp and PCC/precuneus (major nodes of the DMN) was associated with increased CAPS scores (Fig. 2). While stemming from a different section of the DMN, these results align with previous findings (Sripada et al., 2012b) of negative correlation between CAPS scores and functional connectivity between the vmPFC (another node of the DMN) and the hippocampus. The precuneus, located in the medial parietal lobe, is a major hub within multiple brain networks (Utevsky et al., 2014). According to a prominent model of spatial memory (the BBB model, (Byrne et al., 2007)), this region has been dubbed the “parietal window”, operating as an egocentric window into the products of perception, and episodic and spatial memory retrieval, as well as the visual sketchpad upon which visuo-spatial working memory operates. Consequently, the precuneus is crucial for mental imagery, and increased aHipp-precuneus functional connectivity could indicate abnormal recruitment of the aHipp in central DMN functions such as mental imagery, particularly during flashbacks. Interestingly, the pulvinar-precuneus functional connectivity is lower in PTSD relative to controls (Terpou et al., 2018). The pulvinar is a thalamic structure which regulates alpha synchrony and communications between cortical areas (Saalmann et al., 2012). In this regard, we hypothesize that the reduced pulvinar-precuneus and increased precuneus-aHipp functional connectivity may indicate a shift of the precuneal representations, from thalamically-driven sensory-based representations to a heavily emotional memory-based representation scheme, with the aHipp taking on a more hub-like role in the circuit for the storage and retrieval of trauma event memories. Speculatively, the negative correlation between aHipp-precuneus/PCC functional connectivity and CAPS scores could reflect a coping mechanism orchestrated by the traumatized brain to compensate for the impaired emotional regulation circuitry (involving the aHipp) by relying more strongly upon the intact PCC/precuneus (see Akiki et al., 2018), thereby reducing the symptom severity.

### 4.2 Posterior hippocampus and beyond

#### 4.2.1 vmPFC

Our analysis also showed decreased pHipp-vmPFC functional connectivity among those with PTSD compared to controls (Fig. 5). Inhibition of fear is thought to be (at least partially) dependent on hippocampus-vmPFC connectivity (Admon et al., 2013b, Kalisch et al., 2006, Milad et al., 2007), which has been reported to be reduced in PTSD (Admon et al., 2013b). Moreover, the PFC is known to regulate hippocampal processes (Spielberg et al., 2015), and during retrieval of autobiographical memories, there is evidence that the vmPFC drives hippocampal activation (McCormick et al., 2020). Similarly, strong effective connectivity from vmPFC to the hippocampus has been observed during the elaboration phase of emotionally arousing autobiographical memory retrieval (Nawa & Ando, 2020). Furthermore, the hippocampus and vmPFC are principal nodes of the DMN, which plays a major role in episodic memory, internally-directed mental activity and self-related thoughts. Hence, the disrupted vmPFC-pHipp functional connectivity in PTSD could indicate inadequate downregulation of trauma-related hippocampal activation by the vmPFC, which could consequently result in intrusive traumatic memories and impaired episodic autobiographical recall in PTSD (Abdallah et al., 2017, Akiki et al., 2018, Spielberg et al., 2015).

#### 4.2.2 Postcentral/Supramarginal Gyri

A notable finding of this study was the reduced functional connectivity between the postcentral gyrus (primary somatosensory cortex) and the pHipp as well as between the supramarginal gyrus and a/pHipp in PTSD compared to controls. The somatosensory cortex is crucial for detecting touch stimuli and processing self-movement, and the supramarginal gyrus is implicated in bodily self-consciousness and ownership (Bekrater-Bodmann et al., 2014, Rabellino et al., 2020), coding for peripersonal space (Brozzoli et al., 2011), and visuotactile integration (Gentile et al., 2011). The weakened functional connectivity between the hippocampus and areas responsible for processing bodily sensations could partially explain the altered bodily sense and body ownership experienced by those with PTSD (Rabellino et al., 2018a, Rabellino et al., 2018b). In line with this interpretation, the somatosensory cortex was found to be less active in response to non-threatening touch in PTSD (Badura-Brack et al., 2015). The above findings are consistent with the importance of sensory-motor therapies for PTSD (Elbrecht and Antcliff, 2014, McGreevy and Boland, 2020). *Sensory Motor Arousal Regulation Therapy* (SMART) (Warner et al., 2014) is one such intervention; SMART aims to satisfy the sensory-seeking behaviours found in those with PTSD by allowing them to interact with objects that fulfill their need for sensory satiation. This multisensory approach also integrates auditory, visual and tactile information with interactive motor activities. It has been proposed (Harricharan et al., 2021) that sensorimotor interventions for PTSD can ameliorate deficits in emotional self-regulation by re-engaging otherwise “offline” areas such as the prefrontal cortex, which are normally involved in multisensory integration, emotion regulation, and conscious top-down reappraisal. This promotes reintegration of traumatic memories while reducing their negative affect. Based on the results discussed in this study, we hypothesize that the posterior hippocampus may be a critical brain region that is relatively “offline” in those with PTSD and that clinical interventions targeting this region could potentially have enhanced therapeutic efficacy. More specifically, the decreased connectivity observed between the somatosensory cortex and the posterior hippocampus, which contains more detailed contextual representations, might be a prime target for improved sensorimotor interventions that could potentially result in a contextualized sensory representation of trauma memories. In addition, sensory-motor therapies have focused particularly on treating childhood trauma, where trauma memories are often unreachable by verbal recall (Norton et al., 2011). Here, the stimulation of somatosensory and motor pathways may act as a gateway into otherwise inaccessible trauma memories, perhaps by a restoration of the diminished functional connectivity between the hippocampus and somatosensory areas.

#### 4.2.3 Orbitofrontal cortex

In addition, those with PTSD showed increased a/pHipp functional connectivity with the lateral orbitofrontal cortex (lOFC), a brain region associated with obsession, appraisal and moderating reaction to negative affective states (Milad and Rauch, 2007, O’Doherty et al., 2001). It is also activated in anticipation of (Nitschke et al., 2006) and reaction to (Rolls et al., 2003a, Rolls et al., 2003b) unpleasant stimuli (Milad & Rauch, 2007), and in the absence of an expected reward (Milad & Rauch, 2007). In rats, hyperactivation of the lOFC has been shown to impair fear extinction (Chang et al., 2018). Moreover, higher OFC activation is seen in recalling traumatic autobiographical vs. neutral events in both PTSD and control groups, with the PTSD group showing even more OFC hyperactivation (Shin et al., 1999). Thus, increased coupling between hippocampus subregions and the lOFC could explain abnormal fear regulation, a characteristic symptom of PTSD.

#### 4.2.4 Superior temporal gyrus

Furthermore, the superior temporal gyrus (STG) showed increased functional connectivity with the pHipp and especially with the aHipp. STG, the locus of primary and secondary auditory areas (De Bellis et al., 2002, Reale et al., 2007), is the source of the P300 (O’Donnell et al., 1999), an event-related potential (ERP) component elicited by unexpected stimuli (Van Petten & Luka, 2012). Interestingly, combat veterans with PTSD have shown amplified P300 responses when exposed to both trauma-related (Bleich et al., 1996) and novel stimuli (Kimble et al., 2000). Similarly, women with sexual assault-linked PTSD exhibited escalated mismatch negativity, a pre-conscious ERP originating from the auditory cortex in response to a stimulus that differs from a set of identical stimuli (Morgan & Grillon, 1999), aligning with hyper-vigilance often seen in PTSD. Supporting these findings of altered auditory perception in PTSD, one study reported increased STG gray matter volume in children and adolescents with maltreatment-related pediatric PTSD (De Bellis et al., 2002). Another study on those with Acute Stress Disorder found that activity in STG was positively correlated with PTSD severity (Cwik et al., 2017). Taken together with the above, our findings of greater aHipp- and pHipp-STG functional connectivity in PTSD underscore the importance of the STG in the neurocircuitry of PTSD. Furthermore, trauma memories are often accompanied by acoustic components. Thus, it is conceivable that increased hippocampal-STG functional connectivity could reflect this aspect of the trauma memory, especially given that the individuals with PTSD in our sample were combat-exposed war veterans, many of whom would have suffered from exposure to blasts.

#### 4.2.5 Ramification for dual representation theory

Our findings also relate to the Dual Representation Theory (DRT) of PTSD (Brewin, 2014, Brewin et al., 2010), which essentially designates two types of memory that are differentially impaired in PTSD. The first is a perceptual memory system, containing relatively unprocessed and raw sensory and perceptual representations of events *(“S-reps”),* while the second consists of contextualized and verbally accessible representations of events *(“C-reps”)*. S-reps chiefly rely on the dorsal visual stream, the amygdala, and the insula, while the hippocampus and surrounding areas in the medial temporal lobe largely maintain C-reps. Flashbacks are viewed as amplified S-reps that, owing to the extreme stress during the encoding of the traumatic event, are not appropriately paired with the associated C-reps (which themselves are weakly encoded because of the stress), and are hence lacking due context. While the DRT does not posit a role for the hippocampus in flashbacks, our results suggest a refinement of this theory, whereby the aHipp plays a central role in flashbacks. Our finding of increased insula-aHipp functional connectivity is consistent with this, and it would be interesting to explore the directionality of our observed increased functional connectivity between the aHipp and the insula/sensory areas. However, we did not see increased amygdala-aHipp functional connectivity in PTSD, perhaps because they are already strongly connected in the healthy brain. In any case, our findings do not entirely support DRT, as the aHipp is abnormally hyper-connected to affective and multisensory areas in PTSD and is likely to drive trauma memories; this proposition requires further empirical confirmation, e.g., by conducting effective connectivity analyses during both resting-state and tasks involving trauma-related memory recall. Given the extensive and direct connectivity of the aHipp with the amygdala and the insula and the involvement of the aHipp in emotional memory encoding, it is conceivable that trauma memories are over-represented in the aHipp at a ‘‘gist-like'' level while being under-represented in the pHipp, which is thought to contain detailed representations (Poppenk et al., 2013). By this account, trauma-related cues would activate the aHipp, and due to its elevated connectivity with emotional circuitry and sensory areas, the ensuing recollection would be rich in emotional and sensory details. As a refinement of the DRT to incorporate our findings, this would imply improper contextualization of trauma memories, with an over-representation of raw sensory and emotional components in (anterior) hippocampal representations. Conversely, the pHipp would be less involved than normal in retrieving the contextual details of the trauma event memory, aligning with the report that synapses in dorsal CA1 in rodents (analogous to the pHipp in primates) are particularly damaged due to short, concurrent stress relative to ventral CA1 (Maras et al., 2014). This proposal, however, needs to be experimentally confirmed by assessing hippocampal activation and connectivity in individuals with PTSD during trauma memory recall.

Interestingly, we did not find altered hippocampal functional connectivity with the amygdala in those with PTSD compared to controls. As discussed earlier, the findings in the literature surrounding the role of the amygdala in the neurocircuitry of PTSD are mixed (Lee et al., 2021, Schulze et al., 2019, Stark et al., 2015, Suarez-Jimenez et al., 2020, Thome et al., 2019), possibly due to variations in tasks performed during scans. Nonetheless, these discrepant findings hint at a departure from an abnormal amygdala-centric view of PTSD dysfunction. For instance, while Suarez-Jimenez et al. (Suarez-Jimenez et al., 2020) reported occasional amygdalar involvement in some phases of fear conditioning and extinction, they primarily highlight a hypoactive thalamus as a core finding, suggesting it to be the nexus of problematic salience. Collectively, evidence points to more heterogeneous and distributed disruptions in cognitive, behavioural, memory and sensorimotor processes in those with PTSD, which could include both the amygdala and hippocampus.

### 4.3 Limitations and future directions

Although the present results provide valuable insights regarding abnormal hippocampal functional connectivity in PTSD, we were unable to distinguish the dissociative sub-type of PTSD (PTSD + DS) (Lanius et al., 2012). This sub-type afflicts 14–30% of individuals with PTSD and is associated with symptoms of depersonalization and derealization, characterized by experiences of “out-of-body” feelings and/or feelings of themselves or their surroundings as being “dream-like” and not real (Harricharan et al., 2021). It is likely that some participants within this study were from this sub-group. However, we were unable to identify them since the two items addressing depersonalization and derealization in the CAPS questionnaire were not recorded in ADNI. This limitation should be kept in mind when interpreting the presented results since PTSD + DS has a distinct neurological signature compared to PTSD. Evidence suggests that PTSD + DS symptoms originate from excessive top‐down prefrontal inhibition on limbic and brainstem regions (Nicholson et al., 2017). Future work is needed to characterize abnormal hippocampal functional connectivity in the PTSD + DS subtype. Moreover, because the analyses reported here were conducted on previously collected publicly available data, we did not have access to some key details of the scanning conditions, such as the instructions given to the participants or whether they were monitored to prevent them from falling asleep. Additionally, since our participant cohort was comprised of elderly (average participant age 68.3 years), combat-exposed male Vietnam war veterans, our results might not be readily generalizable to females, younger individuals and civilians with PTSD. We particularly caution against generalizing the present results to female populations with PTSD, as a recent study (Helpman et al., 2021) found a significant group-by-sex interaction in the effect of PTSD on functional connectivity between the hippocampus and the precuneus, as well as the hippocampus and the angular gyrus (for a review of sex differences in PTSD, see Seligowski et al., 2020). Furthermore, several participants from the PTSD group in the present study suffered from comorbid conditions such as depressive symptoms, which may have affected our results. Also, while functional connectivity can be built upon structural connectivity, we did not assess structural pathways and therefore cannot determine if the functional connectivity patterns were influenced by their anatomical distances, which could be investigated in future studies. Moreover, rsFC analysis merely estimates the temporal correlation between activations of brain areas and does not reveal the direction of these correlations, warranting further investigation using effective connectivity measures. Finally, rsFC may overlook aberrant activation and functional connectivity patterns that manifest during the performance of specific cognitive tasks such as recalling trauma memories.

#### 4.3.1 Is deliberate retrieval of trauma memories less coherent? Possible role for the pHipp

It has been argued (Bisby et al., 2020) that emotionally arousing and aversive memories, particularly traumatic ones, are less coherent compared to emotionally neutral memories. Three lines of evidence support this view:

1. Normally, episodic memory retrieval is thought to be a holistic, multifaceted phenomenon wherein multiple item-item and item-context associations combine to produce a single “all-or-none” re-experiencing of the event (Horner et al., 2015). Importantly, binding of these multi-modal items together and to the context is thought to be primarily governed by the hippocampus (Cohen & Eichenbaum, 1995).
2. In healthy individuals, negative emotional content differentially impacts memory for sensory constructs versus higher levels of encoding, where the sensory-perceptual encoding of individual items is enhanced at the cost of item-to-item and item-context associations (Bisby & Burgess, 2013). Similarly, the administration of cortisol 30 min before a memory-encoding task decreased item-context associations (Van Ast et al., 2013). Moreover, in healthy individuals, episodic memories with negative content reportedly had lower coherence than neutral memories (Bisby et al., 2018).
3. In those with PTSD, memory deficits extend beyond negative, everyday episodic memories. For instance, their memory for paired associates of emotionally neutral items was reportedly weaker (Golier et al., 2002, Guez et al., 2011), and their allocentric memory processing (which depends on hippocampal functioning (Byrne et al., 2007)) was impaired, while their memories for individual items and egocentric memories remained unaffected (Smith et al., 2015). The adverse effects of high stress on memory were further confirmed by a report of firefighters whose memories concerning the fires they had just fought were more impaired with increasing stress (Metcalfe et al., 2019).

While the above studies have, for the most part, not considered the functional differences between the aHipp and pHipp, recent studies have begun to do so. These investigations suggest that while both regions are involved in encoding spatial context, the posterior hippocampus is more involved in the encoding of fine details and detailed spatial relational information (see, e.g. Nadel et al., 2013). For instance, the ratio of pHipp volume to that of the aHipp was positively correlated with item-context retrieval (Snytte et al., 2020), and the volume of the pHipp mediated between age and spatial context memory performance (Snytte et al., 2022). Moreover, children who performed a colour context encoding task showed recruitment of the pHipp during context encoding whereas those exposed to interpersonal violence had impaired memory of contexts (realistic background scenes) associated with violence (Lambert et al., 2017). Another study reported the recruitment of the pHipp (and the posterior parahippocampal cortex) during retrieval of item-context relations, while the aHipp (and the perirhinal cortex) was activated during retrieval of item-item relations (Sheldon & Levine, 2015).

Considering the evidence on aHipp versus pHipp roles in contextual memory, coupled with memory deficits in PTSD, such as fragmented or incoherent autobiographical memory retrieval, the underperformance of the pHipp (and not the aHipp) might be one of the leading causes of these memory impairments. Arguably, PTSD itself is an adaptive response to trauma exposure, which could manifest as a compensatory over-recruitment of the aHipp in PTSD to support processing of events in threatening situations, coupled with an under-recruitment of pHipp. This hypothesis merits further investigation.

Future studies should examine the differential roles of the anterior and posterior hippocampus in a sample including both PTSD without dissociation and the PTSD + DS sub-type, as well as healthy controls, with a focus on prefrontal-hippocampal functional connectivity. Secondly, to capture the direction of connectivity between the anterior/posterior hippocampus and target ROIs, effective connectivity analyses can be performed using multivariate Granger causality (MVGC) (Barnett & Seth, 2014) and/or Dynamic Causal Modelling (DCM) (Friston et al., 2003). Thirdly, future studies could extend beyond our post-hoc analyses, exploring a wider set of ROIs that could characterize the differential role of the hippocampal subregions in large-scale ROI-to-ROI connectivity in those with PTSD. Finally, it is important to assess activation and connectivity patterns beyond the resting state, particularly during trauma memory recall, as well as in a wider range of participants, including females and those with childhood trauma.

## 5 Conclusion

In summing up our main findings, the current study highlighted aberrations in the functional connectivity of hippocampal sub-regions that could underlie some core symptoms of PTSD. Here, we focused on the anterior versus posterior hippocampus, hypothesizing that they might be differentially affected by PTSD due to their unique connectivity profiles and functional roles. We found that the aHipp is the predominant locus of abnormal functional connectivity in PTSD, showing heightened functional connectivity with many brain regions, including affective areas (i.e., insula, orbitofrontal cortex and temporal pole), sensory areas, and nodes associated with the DMN in those with PTSD. In stark contrast, the abnormal connections of the pHipp were not as numerous as those of its anterior counterpart. Thus, our findings hint at abnormal recruitment of the aHipp in retrieving trauma memories in those with PTSD, while the pHipp might not be as involved in contextual retrieval as it normally should. We also observed decreased functional connectivity between regions responsible for bodily self-consciousness and the anterior/posterior hippocampus, potentially accounting for the altered sense of self and somatosensory symptoms in PTSD. Additionally, our study indicates that disrupted DMN and SN connections, mainly via the aHipp, could be regarded as a neural correlate of PTSD, with the left aHipp taking on a more hub-like role. Finally, the current study also found evidence of a link between reduced symptom severity and increased functional connectivity between the aHipp and PCC/Precuneus, which we speculate could reflect a compensatory mechanism in the brain’s attempt to restore DMN recruitment in memory functions within this altered circuit. These abnormal functional connectivity profiles of hippocampal sub-regions could be predictive of symptom severity and may serve as a biomarker of the disorder. They also have important implications for neuroscientifically-guided therapeutic efforts targeting dysfunctional networks and connectivities, particularly highlighting the advantage of sensory-motor integration therapies for PTSD.

## CRediT authorship contribution statement

**Mohammad Chaposhloo:** Conceptualization, Data curation, Formal analysis, Writing – original draft. **Andrew A. Nicholson:** Writing – review & editing. **Suzanna Becker:** Conceptualization, Funding acquisition, Supervision, Writing – review & editing. **Margaret C. McKinnon:** Funding acquisition. **Ruth Lanius:** Writing – review & editing. **Saurabh Bhaskar Shaw:** Conceptualization, Methodology, Supervision, Writing – review & editing.

## Declaration of Competing Interest

The authors declare that they have no known competing financial interests or personal relationships that could have appeared to influence the work reported in this paper.

## Data Availability

Used publicly available data set
